# An Immunomodulatory Device Improves Insulin Resistance in Obese Porcine Model of Metabolic Syndrome

**DOI:** 10.1155/2016/3486727

**Published:** 2016-10-13

**Authors:** Angela J. Westover, Kimberly A. Johnston, Deborah A. Buffington, H. David Humes

**Affiliations:** ^1^Innovative BioTherapies, Inc., 650 Avis Drive, Suite 300, Ann Arbor, MI 48108, USA; ^2^Department of Internal Medicine, University of Michigan Medical School, 4520C MSRB I, SPC 5651, 1150 W. Medical Center Dr., Ann Arbor, MI 48109, USA

## Abstract

Obesity is associated with tissue inflammation which is a crucial etiology of insulin resistance. This inflammation centers around circulating monocytes which form proinflammatory adipose tissue macrophages (ATM). Specific approaches targeting monocytes/ATM may improve insulin resistance without the adverse side effects of generalized immunosuppression. In this regard, a biomimetic membrane leukocyte processing device, called the selective cytopheretic device (SCD), was evaluated in an Ossabaw miniature swine model of insulin resistance with metabolic syndrome. Treatment with the SCD in this porcine model demonstrated a decline in circulating neutrophil activation parameters and monocyte counts. These changes were associated with improvements in insulin resistance as determined with intravenous glucose tolerance testing. These improvements were also reflected in lowering of homeostatic model assessment- (HOMA-) insulin resistant (IR) scores for up to 2 weeks after SCD therapy. These results allow for the planning of first-in-man studies in obese type 2 diabetic patients.

## 1. Introduction

Insulin resistance is the critical pathophysiological disorder in the vast majority of individuals with type 2 diabetes (T2D). Obesity is the most common cause of insulin resistance. Obesity is associated with tissue inflammation which is now recognized as a critical etiology of insulin resistance [1–4]. Circulating white blood cell counts, including absolute neutrophil and monocyte counts, are elevated in diabetic patients compared to nondiabetics [5–7]. Not only do these cells of the innate immunologic system increase in absolute number, but they also exist in a persistently activated state [8–11]. It is clear that recruitment of circulatory monocytes to form tissue macrophages within adipose tissue is the initiating event in obesity-induced inflammation and insulin resistance [4]. The internal environment of adipose tissue favors the M1 proinflammatory phenotype of adipocyte tissue macrophage (ATM) resulting in tissue inflammation and insulin resistance. Proinflammatory cytokines, produced by ATM and other cells, have been shown to promote insulin resistance in a paracrine and endocrine fashion [12]. Interventions with anti-inflammatory action have beneficial effects to improve insulin sensitivity. Most therapeutic options to date, however, have nonspecific and broad actions to inhibit innate immunologic functions [4]. More specific approaches targeting monocyte/macrophage activity may be a novel intervention to diminish inflammation-induced insulin resistance without interfering with other immunologic activity, thereby avoiding adverse side effects.

The monocyte/macrophage system exists in at least two distinct phenotypes of differentiation: proinflammatory (M1) and anti-inflammatory (M2) [13, 14]. Monocytes are produced in the bone marrow and are continuously released into the circulation, constituting approximately 10% of the leukocyte pool in humans [15]. Human circulating monocytes are not a homogeneous population. Three subsets of monocytes have been identified and are based upon the expression of cell surface markers, CD14 (LPS coreceptor) and CD16 (Fc gamma R111). Within the monocyte population, the majority are the classical subset with high CD14 but no CD16 expression (CD14^hi^CD16^−^), with the minority population further subdivided into the intermediate subset (CD14^hi^CD16^+^) and the nonclassical subset (CD14^low^CD16^++^). The classical and intermediate monocyte subtypes have the ability for phagocytosis and production of inflammatory effectors, similar to the Ly6c^hi^ mouse monocyte. The nonclassical monocytes have a patrolling anti-inflammatory and reparative role similar to the Ly6c^low^ mouse monocyte [15]. Upon inflammatory signals, promoted by infection or tissue injury, circulating monocytes infiltrate tissue and differentiate into either an M1 (inflammatory) macrophage phenotype or an M2 (anti-inflammatory, reparative) phenotype. The M1 monocyte/macrophage is usually the initial responder to coordinate and accentuate the proinflammatory response to destroy invading pathogens and digest cellular and tissue debris. The M2 monocyte/macrophage becomes more prominent later in the process to repair and remodel damaged tissue promoted by this vigorous inflammatory process.

Various chronic organ dysfunction disorders have been associated with chronic inflammation. Chronic heart failure (CHF), chronic kidney disease (CKD), and T2D have been shown to have an increase in proinflammatory CD14^hi^ monocytes compared to normal controls [16–21]. In fact, an increase in inflammatory monocytes in these chronic disease states correlates with worse clinical outcomes along with progressive development of atherosclerosis [16, 17, 20, 22–24]. Proinflammatory monocytes are increased in T2D and correlate with progression to diabetic nephropathy and uremia [18, 21, 22, 25]. Accordingly, a treatment which shifts the circulating monocyte pool to a less proinflammatory phenotype may have a clinical benefit to ameliorate the progression of a chronic inflammatory disorder. In this regard, a biomimetic membrane cell processing device, called the selective cytopheretic device (SCD) [26, 27], has been evaluated. This device, when incorporated into an extracorporeal blood circuit, preferentially binds activated leukocytes including neutrophils and monocytes and in the presence of regional citrate anticoagulation (RCA) immunomodulates the bound leukocytes and releases them back into the systemic circulation. The SCD is similar to a hollow fiber dialysis cartridge but with the blood flow path directed to the outside of the hollow fiber membrane rather than the lumen of the membrane. The blood flow path results in low shear forces similar to capillary shear so the membrane has selectivity to bind activated leukocytes [28]. This continuous cell processing activity results in measurable diminution of excessive inflammatory responses in a variety of acute and chronic disease states. This approach was developed based upon the increasing understanding that inflammation is central to acute and chronic organ dysfunction. To evaluate the effect of the SCD on insulin resistance in a preclinical model of T2D, a series of studies was undertaken as a proof of concept and as a first step to test this approach in the clinical setting.

## 2. Materials and Methods

### 2.1. Large Animal Model of Metabolic Syndrome (MetS)

The Ossabaw miniature swine has been identified as a large animal model for the study of the pathogenesis of MetS. Ossabaw swine, from Ossabaw Island off the coast of Georgia, exhibit a thrifty genotype, which allows them to store large amounts of fat for survival during seasonal famine accentuated by the isolated location of the island. When fed an excess calorie atherogenic diet over several months, Ossabaw swine develop at least 5 of the 6 criteria of MetS, including primary insulin resistance, obesity with significant visceral adipose expansion, hypertriglyceridemia and increased LDL : HDL cholesterol, mild hypertension, and coronary artery disease [29–32].

### 2.2. Protocol for Testing the SCD in an Ossabaw Model of MetS

**Figure 1:**
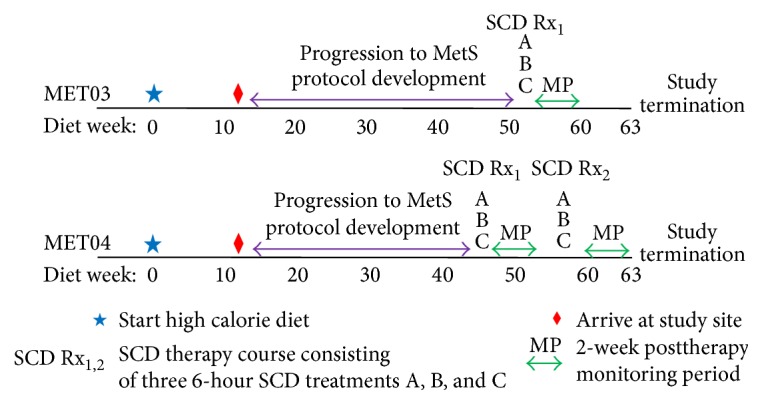
Schematic of the schedule of events for MET03 and MET04 over the progression to metabolic syndrome, SCD therapy protocol development, SCD Rx courses (defined as three 6-hour SCD treatments (A, B, and C) over a one-week period), 2-week posttherapy monitoring periods, and study termination, with respect to weeks on the atherogenic, high energy/high fat diet.

Animal use adhered to principles stated in the Guide for Care and Use of Laboratory Animals (Institute for Laboratory Animal Research, 1996) and procedures were performed under protocols approved by the institutional committee for care and use of animals at the University of Michigan. Two adolescent Ossabaw pigs, procured from Indiana University and identified by MET03 and MET04, were fed an atherogenic, high caloric (4700 kcal/day), high fat diet (KT324, Purina Test Diet, Richmond, IN) to promote obesity and development of insulin resistance [33]. The schedule of events that was followed for the progression to MetS, development of the final study protocol, SCD therapy (SCD Rx) courses, 2-week posttherapy monitoring periods, and study termination for MET03 and MET04 are detailed in Figure 1.

**Figure 2:**
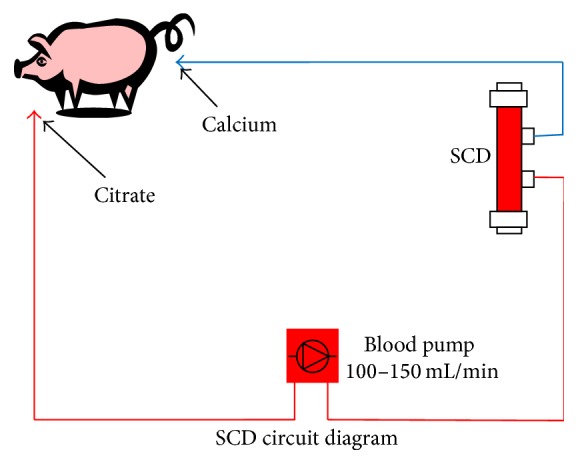
Extracorporeal circuit used to deliver SCD therapy to Ossabaw swine with induced metabolic syndrome.

For surgical placement of cannulas in the external jugular vein, pigs were sedated by intramuscular injection of 2.2 mg/kg xylazine (AnaSed®, Akorn Animal Health Lake Forest, IL) and 6–8 mg/kg Telazol® (Zoetis, Florham Park, NJ) and then anesthesia was maintained with inhalation of isoflurane in 100% oxygen. Excisional biopsy of subcutaneous adipose tissue in the ventral abdominal region was performed concurrently. Postoperative analgesia was provided by transdermal delivery of 20 mcg/hr buprenorphine (Butrans®, Purdue Pharma LP, Stamford CT). Blood sampling and intravenous glucose tolerance testing (GTT) were performed at a minimum of 18 hours postoperatively. Experimental therapy was administered in six-hour treatments using a conventional extracorporeal continuous venovenous hemofiltration (CVVH) circuit, in which the SCD was inserted in place of a standard CVVH hemofilter. Circulating leukocytes were selectively sequestered by the SCD, allowing for the combined effect of membrane interaction in the low ionized calcium environment afforded by the pharmacologic agent, citrate, to inhibit leukocyte inflammatory activity. Citrate also served as the circuit anticoagulant. A double lumen hemodialysis catheter in the jugular vein provided access to and from the animal. Commercial citrate solution (anticoagulant citrate dextrose solution formula A, Baxter, Deerfield, IL) was infused into the extracorporeal circuit, reducing line ionized calcium to 0.25–0.4 mmol/L, which prevented clotting. As per standard RCA protocol, after blood was directed through the extracapillary space (ECS) of the SCD, a 2% calcium chloride solution (American Reagent, Shirley, NY) was infused into the line returning to the animal to prevent a systemic drop in ionized calcium. Systemic ionized calcium levels were measured at least hourly during treatment and maintained in the physiologic range (0.9–1.1 mmol/L) by adjusting the rate of calcium infusion. Blood flow rates through the circuit were targeted at 100–150 mL/min. During therapy, pigs were confined in a crate or sling and sedated by intravenous administration of 0.1–0.5 mg/kg midazolam (BD Rx Inc., Franklin Lakes, NJ) as needed to prevent them from disrupting the circuit. The extracorporeal circuit used to deliver therapy is shown in Figure 2.

Preliminary assessments (data not shown) of impact of SCD therapy on insulin resistance and the inflammatory state in MetS pigs suggested inconsistent outcomes after only one SCD treatment, thereby leading to the final study protocol that encompassed three consecutive SCD treatments (A, B, and C) separated by 24–72 hours (defined as the SCD Rx course). Subsequent to the SCD Rx course, a 2-week posttherapy monitoring period included a customized inflammatory panel, HOMA-IR scoring (see below), and serial GTT to assess the effect of SCD therapy on systemic inflammation and insulin sensitivity. Euthanasia of pigs at study termination was accomplished by intravenous administration of pentobarbital sodium (Euthasol®, Virbac Corp., Fort Worth, TX).

#### 2.2.1. Blood Sampling

All blood samples were collected from an indwelling venous catheter. Sampling included a standard serum biochemistry panel, plasma lipids, plasma triglycerides, total cholesterol, HDL, and LDL. Blood was collected in serum separator tubes and measured by the Marquette General Health System Laboratory (Marquette, Michigan) utilizing colorimetric, potentiometric, and immunoassay methods. Complete blood counts and differentials (CBC) were obtained from whole blood that was collected into sample tubes containing EDTA using an automated veterinary hematology system (HemaVET, Drew Scientific USA). Systemic cytokines were measured in EDTA plasma using commercial, porcine specific ELISA kits (R&D Systems; TNF*α*, PTA00; IL1*β* PLB00B; IL-6, P6000B).

#### 2.2.2. Peripheral Blood Neutrophil and Monocyte Acute Activation Analysis

Samples of peripheral blood were obtained via catheter and immediately placed on ice. Cooled whole blood samples were incubated with fluorescein isothiocyanate (FITC) conjugated antiporcine CD11R3 (ABD Serotec, clone: 2F4/11) and R-Phycoerythrin (PE) conjugated CD14 (ABD Serotec, clone: TÜK4) for 15 minutes, followed by fixation/red cell lysis with BD Facs Lyse solution and analysis via flow cytometry. Porcine neutrophils [34, 35] mobilize intracellular stores of CD11R3 (porcine counterpart to human CD11b) [36, 37] to the cell surface as they become primed for activation, allowing a real time measurement of systemic acute neutrophil (priming) activation. Systemic neutrophil activation can be approximated by determining the ability of the circulating neutrophil population to undergo apoptosis* in vitro*. This was accomplished by isolating neutrophils on a percoll gradient and culturing this purified pool in media containing fetal bovine serum for 24 hours prior to staining with FITC conjugated Annexin V (AbCam, ab14085). Analysis by flow cytometry determined the percentage of neutrophils resistant to apoptosis, reflective of the percent activated neutrophils [38].

#### 2.2.3. Homeostatic Model Assessment of Insulin Resistance (HOMA-IR Score)

Following an overnight fast, a morning blood sample was obtained and assayed for serum glucose and insulin levels. An insulin resistance score was calculated by multiplying glucose (mg/dL) × insulin (*μ*U/mL) and dividing by a constant 405, a formula derived from human medicine [39]. This HOMA-IR value increases as insulin sensitivity decreases.

#### 2.2.4. IV Glucose Tolerance Testing (GTT)

Pigs were fasted overnight. The following morning, pigs were restrained in a low stress sling or a crate to limit activity during the test. Baseline blood samples were obtained from the indwelling catheter, followed by administration of an IV bolus of glucose (500 mg/kg of a 20% dextrose solution, Hospira). Blood samples were collected serially over the next 60 minutes to measure glucose and insulin levels to assess glucose clearance.

#### 2.2.5. Posttreatment Removal of Cells from SCD

At the conclusion of treatment, blood in the extracorporeal circuit was returned to the animal through the circuit using replacement fluid. The SCD was flushed with 1 L of saline and drained by gravity. The SCD was then filled with phosphate buffered saline containing 0.2% EDTA and incubated for 30 minutes at room temperature to release cells that remained associated with SCD membrane. Cells were collected through ECS ports of the SCD after repeated perfusion from lumen to ECS and vigorous perfusion of buffer between ECS ports. Eluted cells were analyzed for total leukocyte counts and differentials to identify the cell populations interacting with the membrane.

#### 2.2.6. Analysis of Adipose Tissue Macrophages

For determination of macrophage tissue infiltration, subcutaneous adipose tissue biopsies were obtained prior to MetS development, during surgery for catheter insertion prior to SCD Rx course and after the SCD Rx course. Adipose tissue was digested as previously described [40] using Liberase (Roche) with the intent of assessing the percentage of macrophages in the stromal vascular fraction (SVF) [41]. SVF cells were classified by flow cytometry (BD Accuri C6) using CD14 (ABD Serotec, clone: TÜK4), CD163 (ABD Serotec Clone: 2A10/11), and CD45 (ABD Serotec, clone: K252.1E4) and verified using manual counts of differentially stained cytospins.

### 2.3. Statistics

 Paired Student's* t*-test, one or two tailed, was used where appropriate with significance set at *p* < 0.05.

## 3. Results

### 3.1. Porcine Model

MET03 and MET04 were started on the high fat, high caloric diet at 2 months of age. Data showed that by month 4 of the special diet these animals had already developed at least 3 of the cardinal features of MetS (central obesity, dyslipidemia, and hypertension) and serial evaluation of glucose and insulin levels revealed a progression toward insulin resistance (Table 1).

### 3.2. SCD Rx Impact on Glucose Tolerance and Insulin Sensitivity

**Figure 3:**
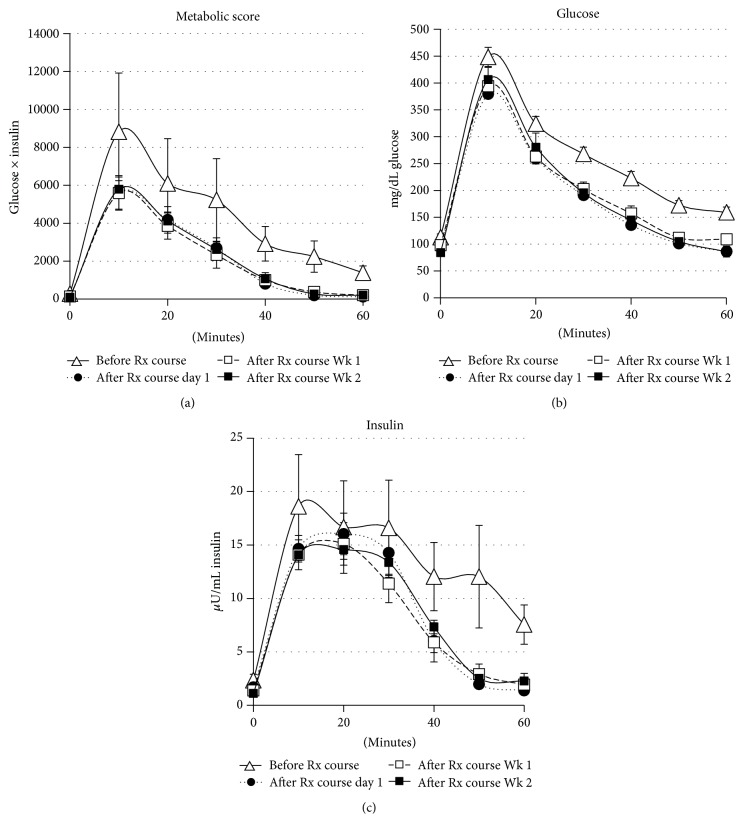
Ossabaw pigs (MET03 and MET04) were started on a high caloric, atherogenic diet at 2 months of age to incite development of metabolic syndrome, including insulin resistance. GTT was performed pre Rx course (∆), day 1 after Rx course (●), week 1 after Rx course (□), and week 2 post Rx course (■). A metabolic score (a), calculated by insulin × glucose levels obtained during GTT, indicated improved insulin sensitivity was conferred by SCD therapy. The effect persisted for 2 weeks after the SCD Rx course. The glucose levels (b) and insulin levels (c) used to generate the metabolic score are also shown. Average of 3 SCD Rx courses that consisted of three 6-hour SCD treatments in one week is shown ± SE (*n* = 3).

A single three 6-hour treatment SCD Rx course for MET03 and 2 three 6-hour treatment SCD Rx courses for MET04 were performed.  Figure 3 depicts glucose levels, insulin levels, and metabolic score (calculated by insulin × glucose) during GTT of both MET03 and MET04 for the two SCD Rx courses that were accomplished with the targeted blood flow rates of 100–120 mL/min and no complications. The baseline GTT curves (∆) indicated that the pigs had progressed to a state of insulin resistance or possibly because metabolic score and glucose remained elevated over the entire evaluation period, T2D. After Rx course, GTT results confirmed an increase in insulin sensitivity was afforded by SCD Rx, and the effect continued for at least 2 weeks.

**Figure 4:**
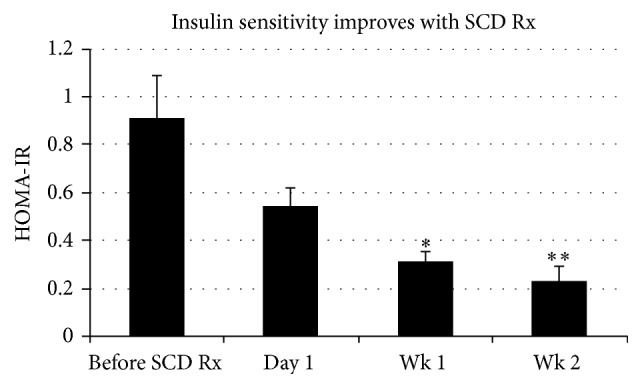
A progressive decrease in HOMA-IR scores reflective of improved insulin sensitivity was observed with SCD Rx. HOMA-IR scores trended lower 1-day post-SCD Rx course and were significantly lower at 1 week and 2 weeks after Rx course monitoring periods. Average of 3 SCD Rx courses that each consisted of three 6-hour SCD treatments in one week is shown ± SE (*n* = 3). *p* = 0.031 (*∗*) and *p* = 0.016 (*∗∗*) for pre-SCD Rx course versus week 1 and week 2 post-Rx course.

HOMA-IR was calculated from collated data collected over each SCD Rx course (Figure 4). A progressive decrease in HOMA-IR scores, reflective of improved insulin sensitivity, was observed with SCD Rx. A HOMA-IR value of 0.6 was determined from preliminary data (not shown) to denote insulin resistance in these animals when compared with GTT curves. Scores dropped below the 0.6 study cut-off value for insulin resistance at day 1 after Rx course and were maintained for at least 2 weeks after Rx course, the latest time point testing being performed.

### 3.3. SCD Rx Impact on Systemic Serum Cytokine Levels

**Figure 5:**
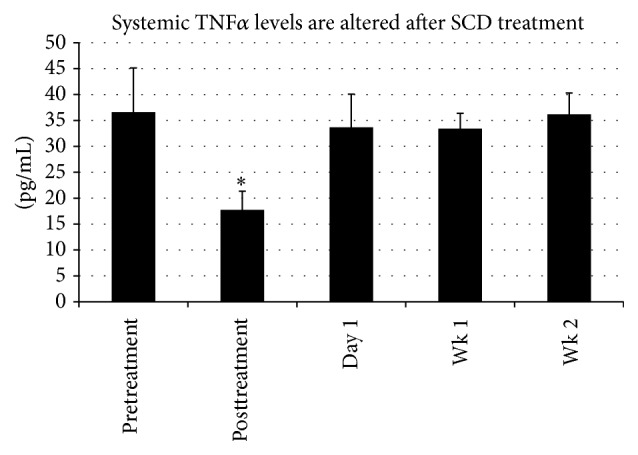
Analysis of circulating levels of TNF*α* was completed for MET03 and MET04 before each treatment (pretreatment), immediately after each 6-hour treatment period (posttreatment), and day 1, week 1, and week 2 following the third treatment. A decrease in TNF*α* was consistently observed from pretreatment to posttreatment, with high significance achieved (*p* < 0.003^*∗*^) when averaging all pretreatment (A, B, and C) levels (*n* = 9) and comparing to all posttreatment (A, B, and C) averaged levels (*n* = 9), providing strong evidence that SCD therapy is able to reduce the chronic systemic inflammation associated with MetS as presented in this model.

Systemic blood samples were analyzed for the cytokines IL-1*β*, IL-6, and TNF*α*. A decrease in TNF*α* was consistently observed over each 6-hour treatment period (A, B, and C), achieving significance at *p* = 0.0022 when all SCD treatment periods A–C were combined (*n* = 9) and providing strong evidence that the SCD Rx course reduced the chronic systemic inflammation associated with MetS as presented in this model (Figure 5). The decrease in circulating TNF*α* was short-lived and on average rebounded within one day after treatment. Systemic IL-6 and IL-1*β* have not been reported in the literature for Ossabaw swine. Upon assay in our laboratory, IL-6 and IL-1*β*, baseline systemic values, were at or below the detection level of the assay.

### 3.4. SCD Rx Impact on Leukocyte Parameters

**Figure 6:**
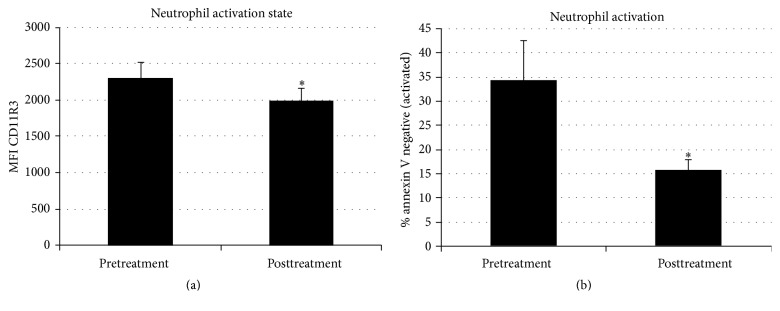
Systemic neutrophil activation can be evaluated by acute changes in CD11R3 expression and the percent circulating neutrophil population that is resistant to apoptosis* in vitro*. CD11R3 expression is increased acutely with neutrophil activation. With SCD exposure, CD11R3 expression decreased (a, *n* = 6 ^*∗*^, *p* = 0.033). During the activated state neutrophil apoptosis is delayed. In these MetS animals, prior to SCD treatment, 34% of neutrophils are activated (resistant to apoptosis), while in healthy animals, less than 10% of circulating neutrophils are expected to be resistant to apoptosis. Analysis of apoptosis, before and after treatments, showed SCD Rx to move the neutrophil population to a less activated state, thus shifting toward naïve percentages of neutrophils resistant to apoptosis (b, *n* = 9, *p* = 0.023).  ^*∗*^For CD11R3 expression, only 2 of the 3 SCD Rx courses with three 6-hour treatments each were included in the analysis (*n* = 6) due to an unanticipated analytic procedural complication.

Prior work has suggested that SCD treatment has critical effects on neutrophils and monocytes [28]. In these animals, the SCD Rx course had a significant effect on circulating neutrophil activation (*p* = 0.033) as measured by CD11R3 and on the apoptotic rate (*p* = 0.023) of circulating neutrophils (Figure 6). Prior to the development of MetS, 90% of isolated neutrophils were apoptotic by 24 hours which translates to neutrophil activation of 10% (resistant to apoptosis).

With the progression to MetS, the percentage of apoptotic neutrophils decreased, indicative of an increased lifespan of activated neutrophils that occurs during a systemic inflammatory process. For both animals, the percentage of activated neutrophils (based on resistance to apoptosis) decreased from a pre-SCD treatment averaged value of 34.4 ± 8.0% to 15.7 ± 2.3% post-SCD treatment (*n* = 9, *p* = 0.023). The decrease in percentage of activated neutrophils persisted through week 1 and week 2 at 17.6 ± 2.0% and 23.4 ± 4.2%, respectively. This decrease in neutrophil activation may be reflective of a shift of the neutrophil population to the shorter life span associated with naïve neutrophils. Absolute neutrophil counts during SCD treatment and through the 2-week followup after SCD Rx showed no change compared the pre-SCD Rx course.

**Figure 7:**
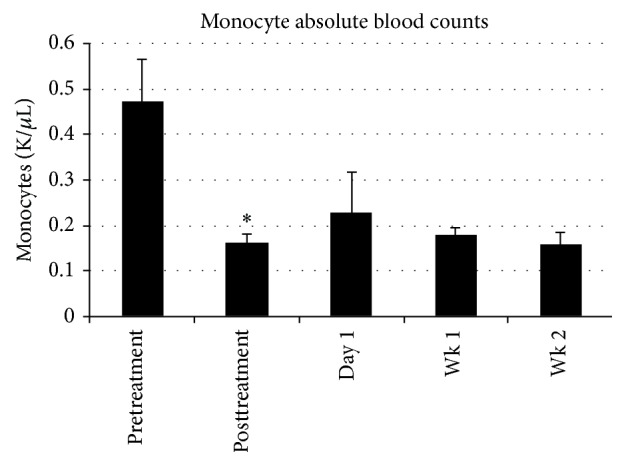
Systemic absolute monocyte blood count was lowered with SCD treatment from 0.47 ± 0.09 (pretreatment) to posttreatment of 0.16 ± 0.02 K/*μ*L (*n* = 9, *p* = 0.007 (*∗*)). This lower monocyte count persisted at the 1-week (0.18 ± 0.02 K/*μ*L) and 2-week (0.16 ± 0.02 K/*μ*L) posttherapy monitoring periods.

For monocytes, SCD treatment reduced the absolute blood monocyte counts from a pre-SCD treatment averaged value of 470 ± 90 to 160 ± 20 K/*μ*L post-SCD treatment (*n* = 9, *p* = 0.007). Monocyte counts were persistently lower compared to pre-SCD treatment levels at 1 week (180 ± 20 K/*μ*L) and 2 weeks (160 ± 20 K/*μ*L) after the SCD Rx course (Figure 7).

### 3.5. Elution of Cells Adherent to SCD

Immediately following treatment, SCD cartridges were eluted and analyzed for total leukocyte counts and differentials in an effort to identify the cell populations that were interacting with the membrane. After 6 hours of SCD treatment, the average number of leukocytes eluted from the 2.5 m^2^ outer fiber surface of the SCD was 7.7 ± 2.2 × 10^7^ cells. Using a cytospin and differential staining, the cells were identified as 70% neutrophils, 23% monocytes, 5% eosinophils, and 0% lymphocytes. With the percentage of neutrophils and monocytes detected in the systemic circulation of these animals typically being ~20–30% and ~3%, respectively, the eluted numbers indicate the preferential sequestration of these cell types, particularly monocytes.

**Figure 8:**
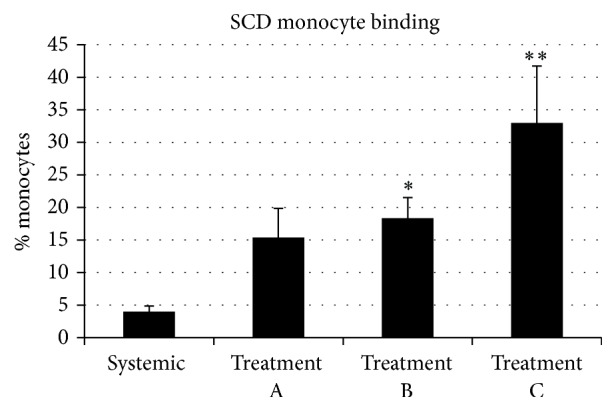
The differential percent of monocytes associated with the SCD membrane increased with each subsequent SCD treatment A, B, and C to 15 ± 4.5, 18 ± 3.2, and 33 ± 8.7%, respectively. For treatments B and C, values are significantly higher than that of the systemic circulation value, prior to SCD treatment, of 4.0 ± 0.9% (*p* = 0.012 (*∗*) and 0.03 (*∗∗*) for B and C versus systemic, resp., *n* = 3).

The differential percentage of monocytes recovered from the SCD membrane was plotted over the three 6-hour treatments (A, B, and C) of each Rx Course. The differential percent of monocytes associated with the membrane increased with each treatment A, B, and C to 15 ± 4.5, 18 ± 3.2, and 33 ± 8.7%, respectively. For treatments B and C, values are significantly higher than that observed in the pretreatment systemic circulation value of 4.0 ± 0.9% (*p* = 0.012 and 0.03 for B and C versus pre-SCD treatment, resp., Figure 8).

### 3.6. SCD Rx Impact on Macrophage Infiltration of Adipose Tissue

In concurrence with other groups that have reported an increase in adipose tissue macrophages [42, 43], in this study, we observed an increase in the percentage of CD14+CD163+ monocyte/macrophage subset in the CD45+ SVF population with the progression of MetS. The absolute number of SVF cells per gram of fat decreased in proportion to the increasing adipocyte size associated with progressive obesity, leading to low SVF cell recovery at final sampling. Due to the small sample size and limited SVF recovery, statistically significant differences were not observed with SCD treatment.

## 4. Discussion

Diabetes mellitus is associated with a chronic inflammatory disorder, especially related to the innate immunologic system with activation of the neutrophil and monocyte populations [5–7]. The initiating event in obesity-induced inflammation and insulin resistance is the recruitment of circulating monocytes to form proinflammatory tissue macrophages within adipose tissue [3]. Persistent activation of circulating neutrophils plays a critical role in progressive microvascular injury in the retina and kidney [9, 44]. Due to this evolving understanding of the role of neutrophils and monocytes in the cause and progressive organ damage of T2D, targeted immunomodulatory approaches to neutrophils and monocytes are being developed to improve insulin resistance and microvascular disease progression in obese T2D patients.

SCD therapy addresses the requirements of this type of immunomodulatory approach for the treatment of T2D. This biomimetic membrane cell processing device is readily incorporated into an extracorporeal blood circuit and preferentially binds activated leukocytes when combined with RCA, followed by release of the immunomodulated leukocytes back into the systemic circulation. This continuous cell processing activity results in measurable diminution of excessive inflammatory responses in a variety of disease states. In preclinical models, SCD therapy has shown efficacy in acute multiorgan injury in severe sepsis. SCD therapy also prevents the inflammatory response to prolonged cardiopulmonary bypass, intracerebral hemorrhage (ICH), and ischemia/reperfusion injury (IRI) and improves myocardial contractility in CHF [45–48]. This device has been tested in 4 clinical studies in ICU patients with acute kidney injury and multiorgan dysfunction requiring dialysis with positive clinical outcomes and no device-related serious adverse events [49–52].

Recent studies have shown that this leukocyte cell processing results in resetting the increased apoptotic life span of circulating neutrophils towards naïve and reducing the circulating levels of the proinflammatory monocyte subtype [53]. This SCD promoted cell processing results in a selective immunomodulating effect on the proinflammatory systemic inflammatory process.

Utilizing a well described porcine model of MetS [29, 32], the SCD was evaluated to assess therapeutic impact to modulate neutrophil and monocyte activity and improve insulin sensitivity before and after treatment in these animals. While rodent models tend to dominate MetS research, the physiological differences between mice and man complicate translation of biomedical research findings into clinical application. Pigs, more similar to humans in many respects, are an attractive alternative, particularly regarding obesity and energy metabolism [54]. A porcine model, such as the obese Ossabaw swine that naturally exhibits many of the features of the metabolic syndrome, may offer better opportunities for translation of the results from promising preclinical investigative research studies into effective prevention or treatment strategies for MetS. Preliminary experiments suggested that a single 6-hour SCD treatment did not have a measurable effect on insulin resistance; therefore, a protocol of three 6-hour SCD treatments (SCD Rx course) over a one-week period was undertaken.

The results demonstrated, as measured with GTT, that, after SCD Rx course, a dramatic improvement of insulin resistance in the animals was observed and persisted for at least 2 weeks, the longest time interval examined in this series of studies. This improvement in insulin resistance was also reflected in the HOMA-IR score in these animals.

Evaluation of neutrophils and monocytes before and after therapy revealed several interesting results. SCD therapy did not change the absolute number of circulating neutrophils but had significant effects to reduce the activated state of systemic neutrophils, as measured by CD11R3 and the percentage of neutrophils resistant to apoptosis. For monocytes, SCD therapy consistently reduced the absolute monocyte counts in blood, with progressively higher differential percentage of monocytes binding to the SCD with each sequential treatment. Preliminary clinical data have shown that circulating proinflammatory monocytes bind more avidly to the SCD membrane [53]. This observation suggests that SCD treatment results in an increased stringency for proinflammatory monocytes binding to the SCD after each sequential treatment. Associated with the reduction in circulating monocyte counts was a significant decline in serum TNF-*α* levels in these animals.

## 5. Conclusions

The results of these experiments have demonstrated that the development of MetS in Ossabaw pigs results in neutrophil and monocyte dysregulation as insulin resistance develops. Treatment with a selective immunomodulating cell processing device (the SCD) promotes a decline in circulating neutrophil activation and monocyte counts in these animals. Associated with these changes is a measurable improvement in insulin resistance. These improved outcomes demonstrated duration of effect over the 2-week posttherapy monitoring period.

This report is limited due to the small number of animals evaluated. This limitation results from the time and expense in developing the MetS syndrome in a large animal model and difficulty maintaining patent vascular access in these research animals over many weeks. Further evaluation of monocyte/macrophage phenotypes is also limited due to the paucity of porcine specific reagents and prior scientific studies to more precisely characterize the various monocyte and macrophage subsets that may be influenced with SCD therapy. In future studies, research effort to evaluate M1/M2 status of monocyte/macrophage subsets in the adipose tissue and circulation will be crucial to the evaluation of SCD therapy.

Despite these limitations, the results obtained in this swine model provide suggestive preclinical data that combined with an excellent safety profile in prior clinical studies with SCD therapy is compelling to consider translation to human studies. In this regard, a pilot early safety/efficacy trial is planned to assess SCD therapy on insulin resistance in obese T2D patients with end stage renal disease on chronic hemodialysis. This patient population is amenable to study due to ease of vascular access for short term extracorporeal therapy and established techniques for human monocyte/macrophage subset classification.

## Figures and Tables

**Table 1:** Biologic, hematologic, and serum biochemical data collected from Ossabaw swine to determine metabolic status. Animals exhibited at least three of the four cardinal features of MetS (central obesity, dyslipidemia, and hypertension) after 4 months on the high fat diet. Insulin resistance cannot be determined from this data due to the fact that values are averaged over each quarter and overlap periods in which pigs were treated with the SCD. Serial glucose tolerance testing (data not shown) indicated a trend toward insulin resistance in both animals.

Biochemical and physical data of Ossabaw swine									
		MET03				MET04			
*Age (months)*		*6*	*10*	*14*	*18*	*6*	*10*	*14*	*18*
*Months on METS diet*		*4*	*8*	*12*	*16*	*4*	*8*	*12*	*16*

Parameter	Mean normal^*∗*^ value								

Weight (kg)	60	43.9	62.7	81.0	90.7	39.8	61.3	78.3	87.3
Abdominal circumference (cm)		96.3	112.0	125.0	168.0	82.0	108.5	122.7	150.0
Blood pressure (mmHg)	88/59	150/94				122/70			
Cholesterol (mg/dL)	73.3	420.0	404.0	499.0	341.3	393.3	379.0	277.0	373.0
Fasting triglycerides (mg/dL)	27.0	43.7	26.5	53.0	38.7	27.7	26.0	25.0	24.3
Fasting glucose (mg/dL)	93.0	137.0	90.5	103.0	92.5	95.7	92.5	113.0	93.3
Fasting insulin (uU/mL)	^*∗∗*^	3.2	1.7	2.2	3.0	1.2	1.0	3.5	1.4
HOMA-IR (glu × ins/405)	^*∗∗∗*^	1.1	0.4	0.6	0.8	0.3	0.2	0.8	0.3

^*∗*^Normal values reported for male Ossabaw swine 12–15 months of age from swine in the laboratory 2nd ed.

^*∗∗*^Normal range has not been determined for fasting insulin using this assay system.

^*∗∗∗*^Since HOMA-IR is a calculation using insulin, a normal range cannot be provided.
